# Chemical Structure, Property and Potential Applications of Biosurfactants Produced by *Bacillus subtilis* in Petroleum Recovery and Spill Mitigation

**DOI:** 10.3390/ijms16034814

**Published:** 2015-03-03

**Authors:** Jin-Feng Liu, Serge Maurice Mbadinga, Shi-Zhong Yang, Ji-Dong Gu, Bo-Zhong Mu

**Affiliations:** 1 State Key Laboratory of Bioreactor Engineering and Institute of Applied Chemistry, East China University of Science and Technology, Shanghai 200237, China; E-Mails: ljf@ecust.edu.cn (J.-F.L.); smmbadinga@ecust.edu.cn (S.M.M.); meor@ecust.edu.cn (S.-Z.Y.); 2 School of Biological Sciences, The University of Hong Kong, Pokfulam Road, Hong Kong, China; E-Mail: jdgu@hkucc.hku.hk; 3 Shanghai Collaborative Innovation Center for Biomanufacturing Technology, Shanghai 200237, China

**Keywords:** microbial biosurfactant, surfactin, structure and property, molecular behavior, potential applications, bioremediation, enhanced oil recovery

## Abstract

Lipopeptides produced by microorganisms are one of the five major classes of biosurfactants known and they have received much attention from scientific and industrial communities due to their powerful interfacial and biological activities as well as environmentally friendly characteristics. Microbially produced lipopeptides are a series of chemical structural analogues of different families and, among them, 26 families covering about 90 lipopeptide compounds have been reported in the last two decades. This paper reviews the chemical structural characteristics and molecular behaviors of surfactin, one of the representative lipopeptides of the 26 families. In particular, two novel surfactin molecules isolated from cell-free cultures of *Bacillus subtilis* HSO121 are presented. Surfactins exhibit strong self-assembly ability to form sphere-like micelles and larger aggregates at very low concentrations. The amphipathic and surface properties of surfactins are related to the existence of the minor polar and major hydrophobic domains in the three 3-D conformations. In addition, the application potential of surfactin in bioremediation of oil spills and oil contaminants, and microbial enhanced oil recovery are discussed.

## 1. Introduction

Biosurfactants are a class of microbial metabolites with surface-active properties and they are capable of spontaneous assemblies at the air–water or water–oil interface and thereby reducing surface/interfacial tensions due to their hydrophilic and hydrophobic structural components. On the basis of their chemical structures, biosurfactants are divided into five major classes of lipopeptides, glycolipids, phospholipids, neutral lipids, and polymeric compounds [1]. Currently, many biosurfactant-producing microorganisms have been isolated and identified to belong to: *Bacillus*, *Agrobacterium*, *Streptomyces*, *Pseudomonas*, and *Thiobacillus* as producers of amino acids-containing biosurfactants; *Pseudomonas*, *Torulopsis*, *Candida*, *Mycobacterium*, *Micromonospora*, *Rhodococcus*, *Arthrobacter*, *Mycobacterium*, *Corynebacterium*, *Mycobacterium*, and *Arthrobacter* as producers of glycolipids; *Thiobacillus*, *Aspergillus*, *Candida*, *Corynebacterium*, *Micrococcus*, and *Acinetobacter* as producers of phospholipids and fatty acids [2].

As one of the typical representative biosurfactants with excellent interfacial and biological activity [3], lipopetides are mainly produced by *Bacillus* (such as *Bacillus subtilis*, *B. licheniformis* and *B. polymyxa*) [4], *Pseudomonas* [5], *Streptomyces* [6], *Aspergillus* [7], *Serratia* and *Actinoplanes*. Lipopeptides are classified as cyclic lipopeptides and linear ones based on their chemical structures [8]. Cyclic lipopeptides refer to those with molecular structure containing a cyclic component formed with carboxyl group in the *C*-terminal of peptide chain bonded to the amino group from the peptide chain or a hydroxyl group from fatty acid chain. Whereas, linear lipopeptides contain a linear amino acid group connected one-by-one and the fatty acid bonded to the α-amino group or other hydroxyl group. Currently, about 90 lipopeptide compounds in 26 families have been identified and most of them were summarized previously by Liu *et al.* [8]. The majority of these lipopeptides belong to the cyclic ones (86 compounds in 24 families), and only four compounds in two families belong to linear ones. Among the 26 families, surfactin [9], lichenysin [10], iturin [11] and fengycin [12] are mostly investigated, of which surfactin is reported so far as the strongest biosurfactant to decrease the interfacial tension of hexadecane/water from 43 to 1 mN·m^−1^ [13,14] and the surface tension of water from 72 to 27 mN·m^−1^ with a critical micelle concentration (CMC) of 10^−5^ M [4,14,15].

This paper presents the primary and spatial structure, property and molecular behaviors of surfactin in aqueous solution and at the interface of oil and water, and its potential application in bioremediation of oil spills and microbial enhancement of oil recovery is also discussed.

## 2. Structure and Property

### 2.1. Structure Characterization

Surfactin was first reported and named in 1968 by Arima *et al.* [9] and its chemical structure was determined by Kakinuma *et al.* [16]. Surfactin composed of a peptide chain formed by seven α-amino acids bonded to a hydroxyl fatty acid by lactone bond to form a cyclic lipopeptide. The typical sequence of amino acids in the peptide ring is: l-Glu^1^-l-Leu^2^-d-Leu^3^-l-Val^4^-l-Asp^5^-d-Leu^6^-l-Leu^7^ [8].

**Figure 1:**
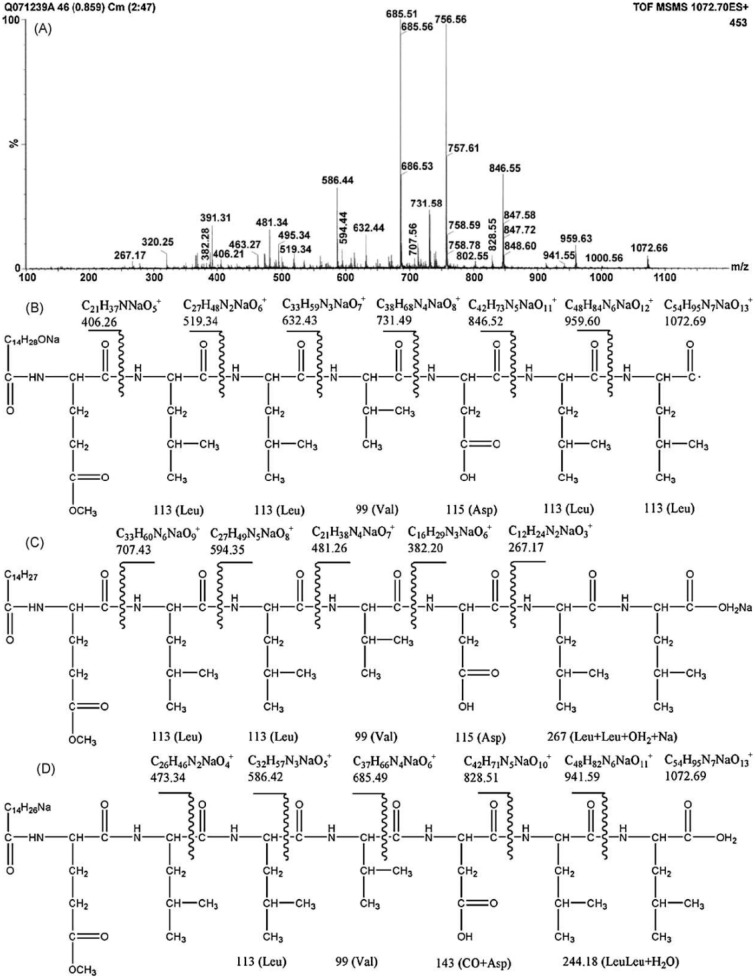
Tandem MS of C15-surfactin-O-methyl ester (**A**) TOF MS/MS; (**B**) Fragment ions after simple cleavage; (**C**,**D**) fragment ions produced after the double hydrogen transfer of lipopeptides in TOF MS/MS. Reprinted from *Process Biochem* 44: 1144 [23]. Copyright (2009), with permission from Elsevier.

**Figure 2:**
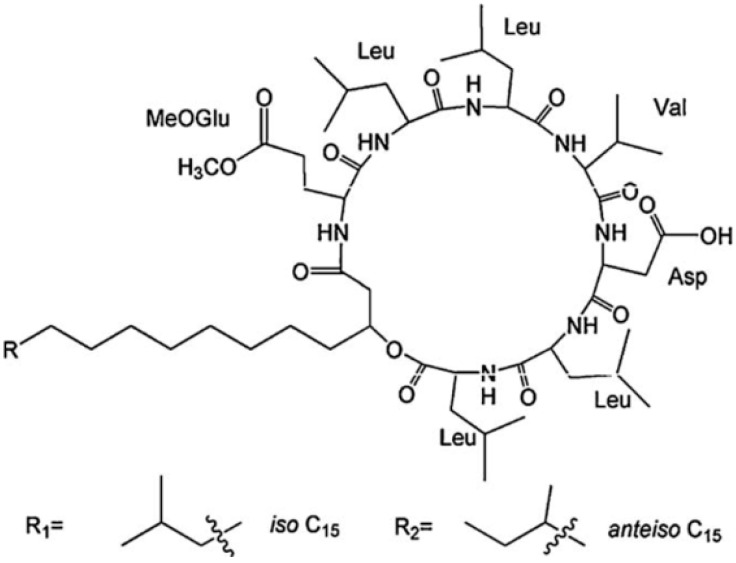
Basic chemical structure of C15-surfactin-*O*-methyl ester. Reprinted from *Process Biochem* 44:1144 [23]. Copyright (2009), with permission from Elsevier.

The differences in the sequence of amino acids and number of carbon atoms in the fatty acids means the surfactins contain a variety of structural analogues [8,16,17]. Baumgart *et al.* [18] reported that three surfactin compounds were produced by *B. subtilis* ATCC 21332 and OKB 105, two of which were different from the basic structure mentioned above in the amino acid Leu^7^ which was replaced by Val and Ile, respectively. Using the method reported by Yang *et al.* [19] for analysis of amino acid sequence and by Yang *et al.* [20] for analysis of fatty acid bonded to the peptide chain, Liu *et al.* [21] identified the surfactins produced by *B. subtilis* when incubated with the same culture medium and found three kinds of surfactins with different peptide moieties: (1) a surfactin with amino acid sequence of *N*-Asp-Leu-Leu-Val-Glu-Leu-Leu-*C*; (2) a surfactin with amino acid sequence of *N*-Glu-Leu-Leu-Val-Asp-Leu-Leu-*C*; and (3) a surfactin with the peptide chain methyl esterified. Meanwhile, the fatty acid moiety of these surfactins was identified to be diverse, including iso C12, iso C13, anteiso C13, iso C14, n C14, iso C15, anteiso C15, n C15, anteiso C16, and anteiso C17 beta-hydroxy fatty acids. Notably, two novel surfactin molecules were isolated and identified in our laboratory in the broth of *B. subtilis* HSO121. One is the C12 lipopeptide [22], which has the shortest chain length among the lipopeptides produced by *B. subtilis*. The other is the C15-surfactin-*O*-methyl ester [23], which is a kind of esterified lipopeptide, approved of biological origin from *B. subtilis* for the first time. The fatty acid portions of C15-surfactin-*O*-methyl ester were identified to be iso C15 and anteiso C15 β-hydroxy fatty acids. The peptide ring was analyzed by tandem MS as shown in Figure 1A, in which the molecular weight of 1049 was determined (deduced from the sodium ionized *m*/*z* 1072.66) and the amino acid sequence of *N*-Glu(OMe)-Leu-Leu-Val-Asp-Leu-Leu-*C* was identified by taking the differences of the fragment ions shown in Figure 1B (simple cleavage of intact lipopeptide) and Figure 1C,D (with double hydrogen transfer). The existence of GluOMe in the peptide ring was further supported by ^1^H NMR (3.66 ppm indicating of the monoester of lipopeptide) and ^13^C NMR (49.15 ppm indicating the methoxy group attached on Glu or Asp). The deduced structure of C15-surfactin-*O*-methyl ester is shown in Figure 2.

Kowall *et al.* [24] also reported that *B. subtilis* produced a total of 44 compounds of surfactins and their monomethyl and dimethyl esters, including the known surfactin variants with l-Leu, l-Val, or l-Ile in position 7 of the peptide ring and unknown variants with leucine residues in position 2 and/or 7 replaced by l-Val and l-Ile.

Different bacterial strains may produce the same surfactin compounds [4], and the same bacterial strains are found to produce lipopeptides belonging to different families as is the case of *B. subtilis* which can produce surfactin, iturin and fenyins simultaneously [25,26,27].

### 2.2. Molecular Behavior at Interface and in Solution

Much attention has been paid to the study of molecular behavior of surfactin at surface/interface because the biological activity mostly occurs at the interface and is governed by the conformational state of molecules at interfaces [28,29]. The processes involved include adsorption, uptake and transport through biomembrane for assimilation and metabolism of nutrients and pollutants. The molecular organization of surfactin at the interface or surfactin conformation at interface have been experimentally investigated or computationally simulated [30].

#### 2.2.1. Interfacial Adsorption

The characteristic of biosurfactant adsorption on the surface and/or interface has attracted more and more attention. The maximum adsorption capacity of 3 × 10^18^ molecule·m^−2^ (as small as 30 Å^2^/each molecule) with surfactin concentration of 3 × 10^−7^ M was detected by Maget-Dana and Ptak [31] which indicate that surfactin molecules at the interface are very closely aligned. Razafindralambo *et al.* [32] studied the adsorption behavior of surfactin homologues by measuring the dynamic surface tension in the surfactin solution phase and found that the adsorption behavior of surfactin from the bulk water phase was not only concentration-dependent, but also influenced by the length of its fatty acid chain. Ishigami *et al.* [15] proposed that surfactin molecules could display dimer formation between two surfactin molecules at the air/water interface. Eeman *et al.* [33] examined the interfacial behavior of cyclic surfactin analogues and concluded that surfactin molecules could rapidly adsorb and organize at the interface after a delay time, which were supposed to be caused by the very low concentration in bulk solution and the large interfacial area in this experiment.

These results show that surfactin is an efficient biosurfactant. The rapid adsorption and the close alignment of surfactin on the surface or interface may be contributed to its specific amphiphilic structure with a hydrophobic moiety consisting of a long fatty acid chain and some lipophilic amino acids (Leu^2^, Leu^3^, Val^4^, Leu^6^, Leu^7^) and a hydrophilic moiety with the backbone of the cyclic peptide and two anionic residues (Glu^1^ and Asp^5^). This characteristic enables surfactin to tend to stay at hydrophilic/hydrophobic interfaces and consequently reduce the surface tension of aqueous solutions, which has great implications for adsorption of hydrophobic molecules, and biological assimilation and transport. From this viewpoint, the longer the fatty acid chain is in the hydrophobic moiety of surfactin, the more intensity there is in the repulsive force between water and this fatty acid chain, and hence the fast adsorption of surfactin to the surface/interface. Gang *et al.* [34] studied the structural and dynamical properties of protonated surfactin molecules at the decane/water interface with molecular dynamics simulation and demonstrated that the peptide rings slightly tilt at the interface and the aliphatic chains exhibited more extended conformation protruding into decane phase, and thus a surfactin molecule would occupy smaller interfacial area. From this viewpoint, surfactin molecules can be closely aligned on the surface and/or interface under certain conditions.

#### 2.2.2. Monolayer at Interface

The amphiphilic property of surfactin leads to the formation of stable insoluble monolayer in the gas–liquid interface. Maget-Dana and Ptak [31] showed that, in the acidic subphase, surfactin monolayer existed in three different physical states classified as: liquid expanded (Le) film, plateau region, and solid film. The appearance of peptide ring and fatty acid chain of surfactin molecules changed with the increment of transition pressure from lying on the gas–liquid interface (in the Le film) to gradually erecting (in the plateau region) and completely perpendicular to the interface (in the solid film), respectively. The same result was also obtained by Ishigami *et al.* [15] except for that the surfactin dimers which were formed due to the hydrophobic interactions between fatty acid chains of surfactin rather than single surfactin molecules behaved at the interface. The subphase temperature and pH may exert influence on the physical states of monolayer in different ways as the subphase temperature alters the mobility of fatty acid chains and pH impacts the ionization of peptide loop in surfactins [35,36,37].

**Figure 3:**
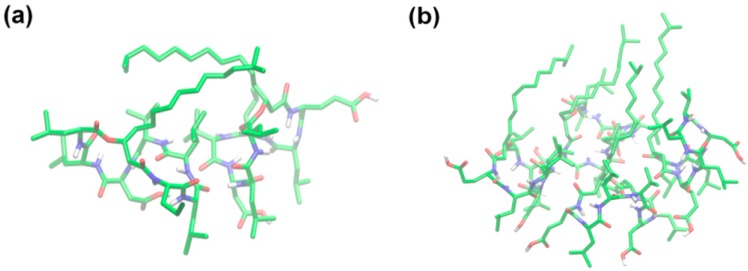
Snapshots of the representative arrangement of surfactin molecules at (**a**) 2.20 and (**b**) 1.20 nm^2^·molecule^−1^ (The atom coloring scheme is C, green; N, blue; O, red; and H, white). Reprinted with permission from Gang *et al.* [38]. Copyright (2011) American Chemical Society.

**Figure 4:**
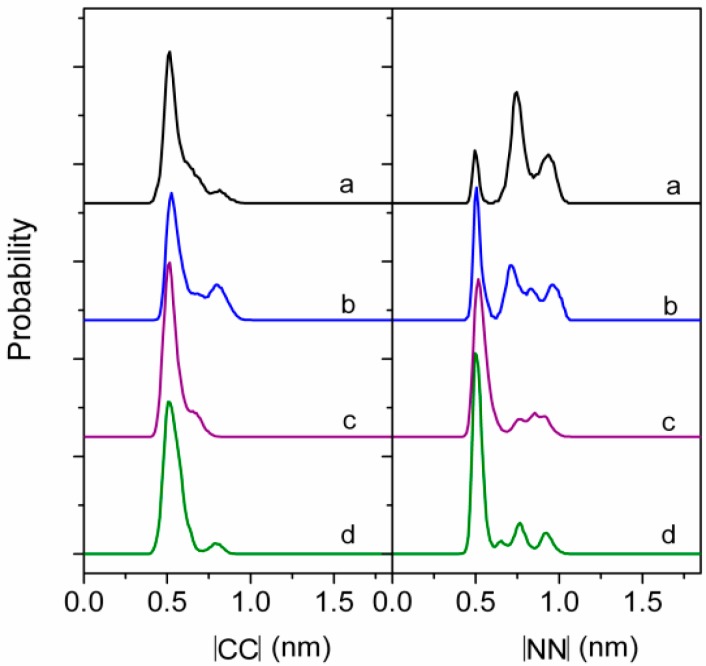
Normalized probability distribution of CC and NN magnitudes (a–d corresponds to the interfacial concentration of 2.20, 1.50, 1.20, and 0.95 nm^2^·molecule^−^^1^, respectively; CC, the vector connecting the β carbon atom of the fatty acid residue and the α carbon atom in Val^4^; NN, the vector connects the nitrogen atom of Leu^2^ and the nitrogen atom of Leu^6^). Reprinted with permission from Gang *et al.* [38]. Copyright (2011) American Chemical Society.

Gallet *et al.* [30] studied the different conformations of surfactin at a hydrophobic/hydrophilic interface and concluded that the peptide ring was positioned in the plane of the interface with the two acidic chains protruding in the aqueous phase, and the β-hydroxy fatty acid chain folded to interact mainly with the side chain of Leu^2^ and also with that of the Val^4^. This display corresponded to the most stable amphipathic structure representing the surfactin experimental behavior under a condition of weak compression. Similar conclusions were obtained by Gang *et al.* [38] who studied the properties of protonated surfactin monolayer adsorbed at the air–water interface with molecular dynamics simulation. Whereas, Gang suggested that the hydrophobic tail was favorable to folding back to interact with the Leu^3^ (not Leu^2^) and Val^4^ residues. Further, she found that the occurrence of hydrophobic contact between surfactin molecules increased with the interfacial concentration, especially the interactions between the hydrophobic tails. The peptide ring backbones exhibited a structural flexibility in that a more packed structure was adopted at a higher interfacial concentration (Figure 3 and Figure 4).

As can be seen from above results, the monolayer of surfactin at interface would be in different physical states at different transition pressures and is mainly influenced by pH and temperature of the subphase. This characteristic presumably benefits the structural distinction of surfactin. The structural flexibility of the peptide ring backbones (as shown in Figure 4), the interactions of hydrophobic fatty acid chain between surfactin molecules as well as the β-folded structure in the surfactin molecule, are all responsible for the transition of monolayer from one physical state to another. The temperature and pH of subphase also influence, to less extent, the characteristics of the surfactin monolayer by enhancing of the activity hydrophobic chain (with increase in temperature) and facilitating the ionization of the carboxylic groups of Glu and Asp (with increase in pH), respectively.

#### 2.2.3. Molecular Behavior in Solution

The molecular behavior of surfactin in solution has been widely studied. Ishigami *et al.* [15] found that surfactin formed large rod-shaped micelles (micelle weight 179,000, aggregation number *n* = 173) having a CMC of 9.4 × 10^−6^ M and γ_CMC_ (surface tension at the CMC) of 30 mN·m^−1^ in 0.1 M NaHCO_3_ solution. Liu *et al.* [39] studied the self-aggregation properties of lipopeptide and found that natural surfactin could form sphere-like micelles and some larger aggregates even at extremely low concentrations. Aggregates in surfactin solution prepared in pH 7.4 phosphate buffer were observed to be of two maximum values in hydrodynamic radius one appeared at 4–6 nm and the other changed with its concentrations as ~85 nm (at 1.0 × 10^−4^ M) and ~108 nm (at 3.0 × 10^−4^ M) [40].

The morphology of surfactin micelle is also affected by pH, metal ions and temperature. Knoblich *et al.* [41] found that surfactin micelle appeared to be spherical, ellipsoidal and/or cylindrical micelles with a non-homogeneous size distribution at pH 7, 9.5, and 12. Furthermore, the cylindrical micelles were transformed into spherical and/or ellipsoidal micelles of smaller sizes by addition of 100 mM NaCl and 20 mM CaCl_2_ to a surfactin solution. The effect of counter-ions (Li^+^, K^+^, Ca^2+^ and Mg^2+^) on micellization behavior was also studied by Li *et al.* [42] and her results showed that the addition of univalent counter-ions tended to form small and spherical micelle particles, whereas, a small amount of divalent cations encouraged the formation of larger micelle aggregates. She *et al.* [43] investigated the influence of ambient temperature on the micelle structure and interfacial properties with Molecular Dynamics (MD) simulations and showed that the surface of micelle with sphere structure shrank and the exposure of the hydrocarbon chains increased with the increasing temperature.

Not only the morphology but also the micropolarity and microviscosity of surfactin micelles are found to be affected by extrinsic factors such as pH, temperature and Ca^2+^. The micropolarity was higher at lower pH and increased in the presence of Ca^2+^ while the microviscosity increased with temperature, especially in the presence of Ca^2+^ [42,44].

**Figure 5:**
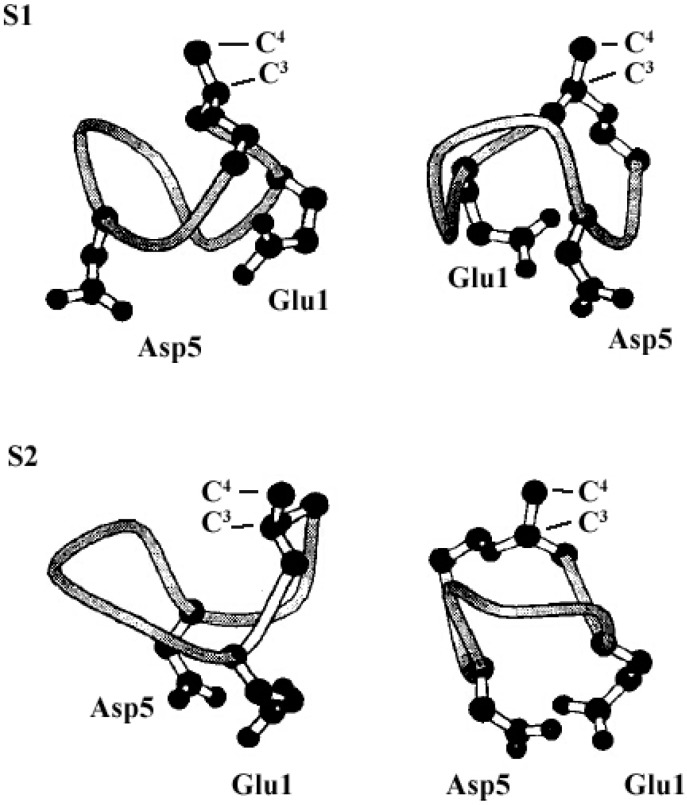
The S1 (**top**) and S2 (**bottom**) structure (only Glu^1^ and Asp^5^ residues have been represented, S1 and S2 exhibit a particular horse saddle folding mode (left) and acidic residues show a claw topology). Reprinted with permission from Bonmatin *et al.* [45]. Copyright (2004) John Wiley & Sons, Inc.

**Figure 6:**
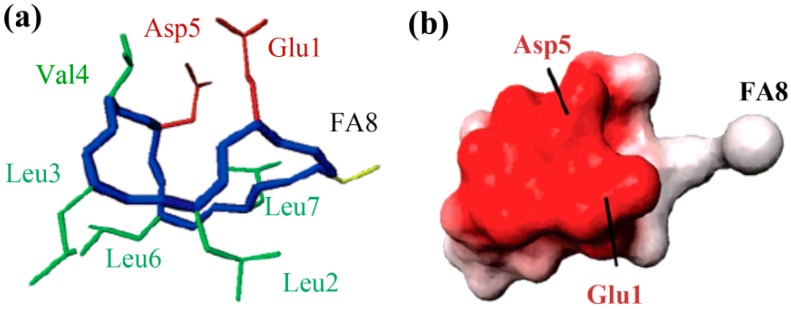
The 3-D structure (**a**) and Electrostatic potentials associated to solvent-accessible surfaces (**b**) of surfactin (The backbone is in blue, the two negatively charged side chains are in red, the FA8 fatty acid in yellow, and the other hydrophobic residues in green). Reprinted with permission from Tsan *et al.* [46]. Copyright (2007) American Chemical Society.

The molecular behavior of surfactin in solution is closely relevant to its spatial structure. Three representative 3-D structures had been developed, among which, the S1 (with a single intra-molecular hydrogen bond, NH(5)–CO(2), fixing a β-turn) and S2 (with three hydrogen bonds, NH(7)–CO(5), NH(4)–CO(2) and NH(6)–CO(carboxyl group), two of them corresponding to γ-turns) were proposed by Bonmatin *et al.* [17,45] with surfactin in DMSO solution on the basis of two-dimensional NMR studies combined with molecular modeling and the other one (with three hydrogen bond, NH(1)–CO(7), NH(4)–CO(1) and NH(5)–CO(1)) was supposed by Tsan *et al.* [46] with surfactin in sodium dodecyl sulfate (SDS) solution. In both S1 and S2 structures, the backbone ring is characterized by a “horse saddle” topology (Figure 5). Residues 2 and 6, which face each other in the vicinity of the acidic Glu^1^ and Asp^5^ side-chains, formed a minor polar domain on one side of the ring and residue 4, lipidic chain, as well as side-chains of residues 3 and 7 constructed a major hydrophobic domain on the opposite side. In the Tsan’s structure (Figure 6), the backbone still adopts a saddle-shaped conformation, with the two acidic residues lying on the same side of the molecule and the hydrophobic residues, except for Val^4^, are all exposed toward the opposite side which formed a negatively charged area surrounded by a large neutral hydrophobic domain (Figure 6). All these three spatial structures, characterized by both hydrophobic and hydrophilic domains in surfactin molecules, are favorable for auto-association and play a fundamental role for the excellent interfacial activity and physicochemical properties of surfactins.

However, based on the comparative Circular Dichroism Spectroscopy (CD) and Fourier Transform Infrared Spectroscopy (FTIR) spectroscopic studies in different solvents with or without Ca^2+^ ions, Vass *et al.* [47] concluded that surfactin had the unique ability of adopting strongly different conformations depending on the ambient conditions.

These results demonstrate that surfactin in solution possess strong self-assembly ability to form micelles and further larger aggregates and the morphology of surfactin micelles formed is notably affected by ambient conditions such as pH, metal ions and temperature.

It is believed that the assembling ability is attributed to the spatial structure with both polar and apolar domains and the β-sheet formation of surfactin molecules [47,48]. The β-sheet conformation, occurring through a hydrogen bond between the carbonyl oxygen from a peptide bond and the other amide hydrogen from the same or an adjacent peptide chain, facilitates the formation of micelles and large aggregates. The formation of micelle and aggregates is further enhanced by the hydrophobic interactions between surfactin molecules with a hydrophobic domain comprising of long chain fatty acids as well as some side-chains of amino residues. The influence of pH on micellization behavior is not fully recognized and the micelle/aggregate morphology is likely not pH-specific [41]. Generally, the breaking of lactone bond and ionization of the two carboxylic groups of Glu and Asp residues in surfactin molecule occurring at high pH are assumed to be important factors affecting the micellization behavior. The addition of counter-ions would impact the appearance of micelles and aggregates, as supposed, in two ways: (1) Shielding the charge of anions of carboxylic groups which may reduce the substantial surface area of head groups in micelles [41]; (2) Constructing a intermolecular salt-bridge as suggested by the formation of a lichensyin-Ca^2+^ complex in a molar ratio of 2:1, thus facilitating micellization via a dimer assembly, with a possible long-range effect on the spatial structure and morphology of the micelles [49]. Temperature also influences the morphology of surfactin micelles and aggregates, as temperature increases, the stability of the hydrogen bond in the micelles decreases and the dehydration occurs and thus the shape of the peptide rings becomes planar, the solvent accessible surface area decreases and, under high temperature, some hydrocarbon chains reversed their orientation. All these factors consequently induced the changes in the morphology of surfactin micelles and aggregates [43].

### 2.3. The Structure–Property Relationship

Surfactin exhibits a strong surface activity by reducing the surface tension of water to 27 mN·m^−1^ with a CMC of 10^−5^ M and is the most effective biosurfactant in terms of fundamental dynamic and equilibrium interfacial properties [3]. In addition, surfactin possesses excellent foaming properties to produce and stabilize foam with higher maximum density and stability at a concentration as low as 0.05 mg/mL [50].

**Figure 7:**
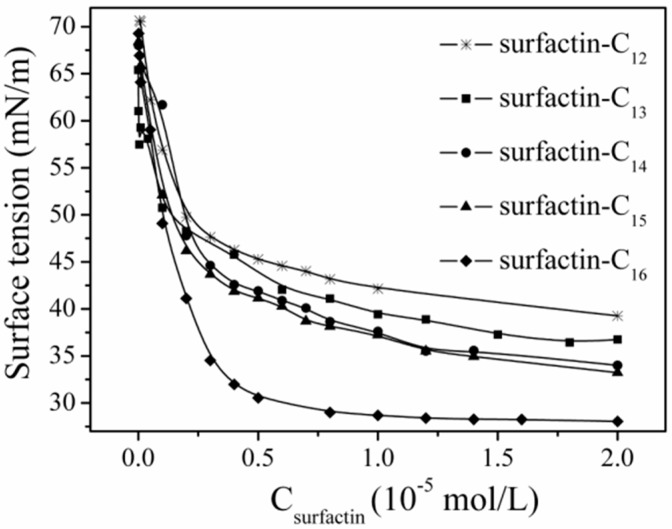
Surface tension of surfactins with different fatty acid chain length [51].

**Figure 8:**
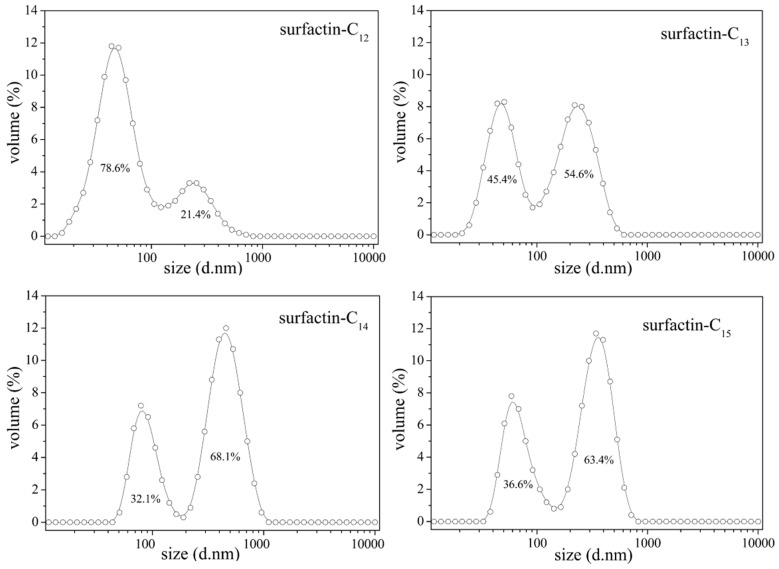
Micelle size distribution of surfactin-C13, -C14 and -C15 [51].

The differences in structure, the carbon length and structure of fatty acid, amino acid composition as well as their sequence in the peptide chain, have some specific impact on the property of surfactins. Generally, surfactins showed a stronger ability in reducing both the dynamic and equilibrium surface tensions with more hydrophobic alkyl chains [32,33,51] and this was typically shown in Figure 7. Also, this phenomenon was found with lichenysin A, another efficient biosurfactant belonging to lipopeptide with a similar primary structure to surfactin of cyclo-(l-Gln^1^-d-Leu^2^-l-Leu^3^-l-Val^4^-l-Asp^5^-d-Leu^6^-l-Ile^7^-β-OH fatty acid). Surface tension of lichenysin A solution were found to decrease with the increase in the percentage of branched chain fatty acids and increase when the percentage of straight chain 3-hydroxy tetradecanoate increased [52]. With regards to the molecular behavior of surfactin in solution, the micellization and aggregation of a group of surfactin isoforms with different fatty acid chains in water solution were studied by Li *et al.* [51] and the results indicated that surfactin with short chain of fatty acids tended to form small micelles, and that with chain of long fatty acids tended to form larger micelle aggregates (Figure 8).

Linear surfactin shows lower surface activities and poor hemolytic activities compared to those of cyclic structures [53]. Eeman *et al.* [33] found that the opening of the peptide ring reduced the surface activity. Morikawa *et al.* [54] demonstrated that the oil displacement activities of linear surfactin, prepared by saponification of the lactone ring, decreased to one third of its parent cyclic surfactin. This difference confirmed the hypothesis that the “horse saddle” structure of surfactin with distinct polar and apolar domains played an important role in surfactin properties. Interestingly, surfactin-C15 conformers were found to possess the same oil displacement activities even though they showed different minimum surface tensions [54]. These results implied that surfactin conformers would have different structures in water, but the same structure at the water–oil interface.

The two Glu and Asp residues present in the cycle of surfactins play a critical role in their amphiphilic properties. Liu *et al.* [23] studied the surface activity of C15-surfactin-*O*-methyl ester (Figure 2) with comparison of C15-surfactin and found that C15-surfactin-*O*-methyl ester had a stronger surface activity than C15-surfactin. C15-surfactin-*O*-methyl ester showed CMC of 9.78 × 10^−6^ M and surface tension at CMC (γ_CMC_) of 28.71 mN/m in 10 mM phosphate buffered saline (pH 8.0, 25.0 °C), whereas, C15-surfactin was characterized with CMC of 1.74 × 10^−5^ M and γ_CMC_ of 30.03 mN/m under the same conditions. Morikawa *et al.* [54] found that the activity increased by 20% when the Glu and Asp residues were both methylated or amidated, and decreased drastically when these two residues were modified by aminomethane sulfonic acid. Thimon *et al.* [55] studied the surface properties of the chemically modified (Glu γ-methyl ester) surfactin and found this variant showed a lower CMC than the mother surfactin. It was also confirmed that the surface activity of monoanionic biosurfactants (e.g., lichenysin A with asparagine in position 5) was higher than that of dianionic biosurfactants (e.g., surfactin with aspartate in position 5) [56,57]. Grangemard *et al.* [49] conducted a comparative study on the properties of lichenysin produced by *B. licheniformis* with the surfactin produced by *B. subtilis* (with similar primary structure but different in Glu^1^ and Leu^7^ in surfactin, Gln^1^ and Ile^7^ in lichenysin) and found that lichenysin was with a higher surfactant power (CMC, 22 μM) than surfactin (CMC, 220 μM).

In terms of the role of amino acids in the modulations of activities, Bonmatin studied the properties of surfactin modifiers with the substitution of the L-valine^4^ by a more hydrophobic residue, *i.e.*, leucine or isoleucine and found that these Leu^4^- and Ile^4^-surfactins had a higher affinity for hydrophobic solvents and a twofold decrease of CMC [58]. In light of the three-dimensional structure, the residue 4 side-chain was proposed to be aligned along the direction of the lipid tail, so it reinforces the hydrophobic side and, hence, plays a critical role in surfactant activity. Also, experiment results showed that the surface activity was increased and CMC decreased when the amino acids of surfactin were changed to more hydrophobic ones [4,58,59].

The ambient pH also affects the surface/interface property of surfactin markedly. Surfactins show higher activity under alkaline conditions than under acidic conditions. When aspartic acid residues and glutamic acid of surfactins were amido-esterified or methyl-esterified, the obtained derivatives retained similar activities irrespective of the pH changes [54].

Surfactin, with β-hydroxy fatty acids linked to a heptapeptide (l-Glu^1^-l-Leu^2^-d-Leu^3^-l-Val^4^-l-Asp^5^-d-Leu^6^-l-Leu^7^), obviously presents the amphiphilic character. Based on all experimental data mentioned above, the three-dimensional structures proposed by Bonmatin *et al.* [45] and Tsan *et al.* [46], characterized by the formation of both the hydrophilic domain and hydrophobic domain, substantially account for the activities of surfactin. This was confirmed by two facts: (1) the fatty acid chain plays a fundamental role in formation of supramolecular structures such as micelles or “oligomers” through intermolecular hydrophobic interactions; (2) the carboxylic groups of both glutamate and aspartate, the polar domain, in surfactin molecules in the aggregation process, plays the predominant role in the properties of surfactin. It would be more reasonable to conclude that the solubility and surfactant activities of surfactin depend on the precise distribution of the residues forming the two respective domains.

## 3. Potential Applications

During the last few years, there has been an increasing demand for biosurfactants used as emulsifiers, de-emulsifiers, wetting agents, spreading agents, foaming agents, functional food ingredients and detergents. Recently, due to several major advantages over chemical surfactants—lower toxicity, high environmental compatibility, biodegradability, and synthesis from renewable raw materials—biosurfactants have been becoming the focus of extensive research and applications from food industries to oil industries.

### 3.1. Microbial Enhanced Oil Recovery (MEOR)

MEOR is considered as a tertiary oil recovery technology that uses microorganisms and their metabolites to mobilize residual oil in oil reservoirs which is trapped in porous rocks by capillary force [60]. Due to the fact that capillary force is believed to be proportional to the interfacial tension (IFT) between oil and water and that biosurfactants are effective in reducing capillary forces by the reduction of oil–water interfacial tension and wettability alteration [61,62] and in forming micelles, biosurfactants, thus, possess the capability to facilitate mobilization of the entrapped oil in reservoirs [1,63].

Recent studies have shown that IFT reduction and wettability alteration are important mechanisms for MEOR [64,65]. Further, Sarafzadeh *et al.* [61] conducted several different tests to quantify the individual effects of the IFT reduction and wettability alteration on the efficiency of MEOR using *E. cloacae* and concluded that wettability alteration played a more significant role in improving oil recovery for both *ex situ* and *in situ* scenarios than IFT reduction. In addition, Sarafzadeh’s results showed that wettability alteration was changed mainly by biosurfactant adsorption on the concerning rock.

Various experiments with laboratory-scale of sand-packed columns and field trials have successfully demonstrated the effectiveness of biosurfactants in MEOR. Use of biosurfactants in MEOR can be implemented in two different ways as either an *ex situ* biosurfactant injection or *in situ* biosurfactant production to achieve increase in oil recovery from subsurface reservoirs [66]. Both of them require that biosurfactants and their producing microorganisms are able to tolerate the harsh environmental conditions, such as high salinities, temperatures, and pressures. Possessing extremely advantageous features, including lower toxicity, higher biodegradability and effectiveness at extreme temperature, salinity and pH conditions as well as high interfacial activities together with low CMCs, surfactin is the most promising candidate in MEOR [66].

Surfactins can maintain activities under harsh conditions and recover sand trapped oil. Al-Wahaibi *et al.* [67] reported that surfactin from isolate B30 could form stable emulsions with light and heavy crude oil, and enhance oil recovery by 17%–31%. Meanwhile, this surfactin was proved to be stable over a wide range of pH, salinity and temperatures [67]. Schaller *et al.* [68] concluded that surfactin was active under harsh conditions with NaCl concentration of 0%–10%, pH 3–10 and temperature of 21–70 °C. Makkar and Cameotra [69] found that *B. subtilis* produced surfactin at high temperatures which could emulsify diesel with 90% E_24_ and recover 62% of oil entrapped in sand core. All of the above results show the potential of surfactins for MEOR.

Biosurfactant-producing microorganisms can survive and produce biosurfactant in *in situ* conditions of oil reservoirs. By inoculation to oil reservoirs with *Bacillus* strains and/or nutrients, Youssef *et al.* [70] confirmed that the *Bacillus* strains tested could produce lipopeptides with an average concentration of 90 mg/L in the oil reservoir, which was approximately nine times the minimum concentration required to mobilize the entrapped oil from sandstone cores. Besides, the microbial metabolism and *in-situ* biosurfactant production as well as concomitant increase in oil production were observed and, thus, the metabolic activity of injected biosurfactant-producing microorganisms was confirmed [71].

In the past decades, about 10 MEOR applications have been implemented in Malaysia, USA, Argentina, and China and a successful MEOR field test was observed with oil reservoir temperatures below 55 °C, salinity less than 100,000 ppm, water cut above 75%, and a low production rate [72]. Maudgalya *et al.* reviewed 26 biosurfactant-related field trials of MEOR and found that 20 of them (77%) were positive in oil production [73]. Till now, one of the most encouraging results of MEOR was found in Shengli oil field, China, with production of 9.75 × 10^4^ t additional crude oil over 10 years [74]. With almost a century of research and field trials, MEOR has shown great potential in enhancing oil production and extending the economic life of an oilfield.

### 3.2. Oil Spill Contamination and Remediation

Oil spills are one of the major causes of oil pollution in marine environments. Due to the toxic and persistent characteristics, chemically synthesized surfactants are not favored as remediating agents.

Biosurfactant is a potential alternative for bioremediation of crude oil contamination and oil spills in marine environments and other terrestrial ecosystems for its excellent surface activity and emulsifying properties as well as lower CMC characteristics. It has been shown that from the above CMC, stable aggregates are formed (called micelles) which can increase the solubility of hydrophobic organic contaminants, providing opportunities for phyto- or microbial remediation. The ability of biosurfactants in emulsifying hydrocarbons into water to form mixtures has been widely reported [75]. These emulsification properties are believed to facilitate hydrocarbon release, solubilization, assimilation and degradation by flora and fauna in the environment, the very fundamentals for oil spill cleanup [76]. Several biosurfactants have found successful applications in environmental remediation such as rhamnolipids [77], lichenysins [78] and surfactin [14,79]. Kim *et al.* [80] isolated a new bacterial strain from a crude oil sample collected in an oil spill contaminated area, and found that the biosurfactant produced by this strain showed good emulsification activities on paraffin and crude oil. The addition of biosurfactant-containing remediation agent to seawater stimulated the degradation of crude oil by the indigenous marine bacteria as well as the removal of crude oil from the surface of contaminated sea sand [81]. Meanwhile, biosurfactant is capable of disrupting oil slicks floating on the water surface, promoting oil dispersion, generating stable emulsion and thus greatly enhancing its biodegradation by indigenous marine microorganisms [82] as well as enhancing the attachment of bacteria to oil layers to increase the uptake and metabolisms of oil hydrocarbons [83]. These factors indicate that biosurfactants have a strong potential for applications in cleaning up oil spills at sea and on shorelines.

Biosurfactants produced by one consortium of microorganisms may enhance the biodegradation of oil hydrocarbons by other microorganisms. Considering the ubiquitous existence of marine hydrocarbon-degrading bacteria—the obligate hydrocarbonoclastic bacteria (OHCB) which have been recognized and shown to play a significant role in the biological removal of petroleum hydrocarbons from polluted marine waters [84,85,86]—biosurfactant added to or produced *in situ* in the contaminated environment will be of great significance in the oil clean up. Laboratory studies have shown that the addition of biosurfactant mixtures alone is useful for stimulating biodegradation of hydrocarbon contaminants in the environment [87]. The wide range of hydrocarbonoclastic capabilities of bacterial consortium leads to the degradation of both aromatic and aliphatic fractions of the crude oil [88]. The effectiveness for *in situ* bioremediation of oil contaminated soil by biosurfactant-producing *B. subtilis* was also demonstrated [89].

Generally, oil-degrading microorganisms have the capacity to produce biosurfactants to facilitate their growth by enhancing assimilation of hydrocarbons and nutrients available from the environment. Even in the cold oceanic environment, some oil-degrading strains can thrive and degrade oil normally and the low temperature had no obvious effects on the hydrocarbon degrading bacteria community and the bioremediation process [90]. A number of hydrocarbon-degrading microorganisms are reported to produce emulsifying agents and some of these bioemulsifiers have been considered for use in oil cleaning operations and other applications at low temperature. 

Biosurfactants were experimentally investigated for their ability in facilitating the removal of adherent oil from Alaskan beaches and results demonstrate that microbial surfactants released oil to a significantly greater extent (two to three times) than water alone [91]. The ability of dispersing oil slicks into fine droplets, converting mousse oil into oil-in-water emulsion and washing oil away from oil entrapped sand has been shown with convincing results [87]. Due to the lower CMC than their chemical counterparts, biosurfactants are the suitable candidate for use in oil spill treatments and mitigation.

The results of field experiments and trials following actual spill incidents have been reviewed by Swannell *et al.* [92]. The oil-degrading microorganisms in the marine environment are well established and research has demonstrated the capability of the indigenous microflora to degrade many chemical components of the petroleum shortly after exposure. Application of fertilizers can also effectively stimulate the biodegradation rates of oil. The ratio of oil loading to nitrogen concentration is considered to be the principal controlling factor of this bioremediation strategy in field.

Evaluation methods have been established [93]. Dispersion effectiveness of surfactins was evaluated by measuring the CMC and dispersant-to-oil ratio (DOR). For surfactins, the CMC values in double deionized distilled water were lower than that in the sea salt solution, which has been partly attributed to saline-induced conformational changes in the solvated ionic species of the biosurfactants. The DORs for surfactin were 1:96 in water, much higher than that in 12 ppt sea salt of 1:30, suggesting a reduction in oil dispersing efficiency of surfactant in saline.

### 3.3. Other Applications

Being amphiphilic membrane active biosurfactants, surfactin, fengycin and iturin compounds are found to be of potent antimicrobial activities. Iturin are potent antifungal agents which can be used as biopesticides for plant protection. Iturin A produced by *B. subtilis* RB14 was shown to be effective for the control of damping-off caused by *R. solani* in tomato plants [94]. *B. subtilis* 20B showed antifungal activity against mycelia growth of *C. indicum*, *A. burnsii*, *F. oxysporium*, *F. udum*, *T. herzanium* and *R. bataticola* [95]. Surfactin of *B. subtilis* have the most potent antibiotic activity [4] and that from *B. licheniformis* inhibits germination of *M. grisea* [96]. Bechard *et al.* [97] demonstrated that lipopeptide from *B. subtilis* possessed a broad spectrum of activity against Gram-negative bacteria, little activity against Gram-positive organisms, as well as one fungi assayed.

Taking into consideration the amphiphilic nature of these lipopeptides, the above-mentioned activities are presumably believed to be a direct consequence of the interaction of lipopeptide with its target membrane and the alteration of the bilayer properties. The interaction of lipopeptides with membrane lipids may form a pore which causes cellular leakage [98,99]. Carrillo *et al.* [100] observed that the incorporation of surfactin into POPC membranes induced a strong dehydration of the phospholipid C=O groups and a strong interaction with the phospholipid acyl chains and all these interactions finally result in loss of vesicular contents, or “pore” formation which consequently induces content leakage. It was believed that cyclic peptides killed Gram-positive and Gram-negative bacteria in this way [101].

Also, biosurfactants have found application or prospect in cosmetics [102], the food industry [103], the environment [104,105,106], pharmaceuticals [6,7,107], and biological control of harmful microorganisms in agriculture [108,109].

## 4. Conclusions

Biosurfactants are a unique class of microbial metabolites with surface-active properties. The differences in the sequence of amino acids and carbon atoms in the fatty acids provide surfactins with diverse chemical structures and physiochemical properties. The amphiphilic characteristics of surfactin molecules enable them to have a strong tendency to adsorb to hydrophilic/hydrophobic interfaces and, consequently, reduce the surface tension of aqueous solutions, leading to the formation of a stable insoluble monolayer in the gas–liquid interface. The physical states of this monolayer are influenced by subphase temperature and pH. The fact that surfactins exhibit a strong self-assembly ability to form micelles and some larger aggregates, even at rarely low concentrations, can be reasonably explained by these three typical 3-D structures that are developed. The amphiphilic and surface properties of surfactins are certainly related to the existence of the minor hydrophilic and major hydrophobic domains as described in the three 3-D conformations. Recently, due to several advantages over chemical surfactants such as lower toxicity, high environmental compatibility, biodegradability, and synthesis from renewable raw materials, biosurfactants have been the focus of extensive research and applications, especially in microbial enhanced oil recovery as well as remediation of oil spills.
